# Phytomedicine in Disease Management: In-Silico Analysis of the Binding Affinity of Artesunate and Azadirachtin for Malaria Treatment

**DOI:** 10.3389/fphar.2021.751032

**Published:** 2021-11-30

**Authors:** Michael P. Okoh, Rajeev K. Singla, Chijioke Madu, Opeyemi Soremekun, Johnson Adejoh, Lukman A. Alli, Bairong Shen

**Affiliations:** ^1^ Institutes for Systems Genetics, Frontiers Science Center for Disease-related Molecular Network, West China Hospital, Sichuan University, Chengdu, China; ^2^ Department of Medical Biochemistry, Faculty of Basic Medical Sciences, College of Health Sciences, University of Abuja, Abuja, Nigeria; ^3^ The African Computational Genomics Group, MRC/UVRI at London School of Health and Tropical Medicine, Entebbe-Uganda, United Kingom; ^4^ Molecular Bio-computation and Drug Design Laboratory, School of Health Sciences, University of KwaZulu-Natal, Westville Campus, Durban, South Africa

**Keywords:** malaria, phytomedicine, gephyrin, metabolite, molecular dynamics, protease, reactive oxygen species, SDICS methodology

## Abstract

In the rural communities of sub-Saharan African (sSA) countries, malaria is being managed using phytocompounds. Artesunate is reported to inhibit Gephyrin E, a central, multi-domain scaffolding protein of inhibitory post-synapses. Neem plant and its metabolites like azadirachtin are being indicated for management of malaria by traditional healers. The present study was aimed to cheminformatically analyse the binding potential of artesunate and azadirachtin with various reactive moieties of Gephyrin E, to reduce malaria scourge. With molecular dynamics (MD), binding free energy estimation and binding affinity of artesunate and azadirachtin to Gephyrin E was done. GRIP docking was done to study the interactions of these test ligands with Gephyrin E (6FGC). MD simulation gave insights to structural changes upon binding of artesunate and azadirachtin in the ligand-binding pocket of Gephyrin E. Root mean square deviation (RMSD) and root mean square fluctuation (RMSF) were calculated. From the estimation, azadirachtin had a total binding energy of −36.97 kcal/mol; artesunate had a binding energy of −35.73 kcal/mol. The GRIP docking results provided a clearer evidence that artesunate has comparatively better binding affinity to Gephyrin E than azadirachtin, and the critical binding sites (in activity order) were cavity 3, 2, 8, and 6 for artesunate while for azadirachtin, it was cavity 6, 3, 8, and 2. The GRIP docking provided detailed interactions at the atomic levels, providing evidence; both compounds have chances to overcome the drug resistance problem, albeit higher for artesunate. Our findings added another piece of evidence that azadirachtin may be effective as an anti-malarial agent. The results herein may provide impetus for more studies into bioactive components of plant origin towards the effective management of malaria disease phenotype.

## Introduction

There is an increase in the epidemiological burden of severe life-threatening diseases on the human population across the globe. This has compelled researchers and clinicians to develop reliable therapeutic strategies against these diseases (Srivastava et al., 2019). Different disease conditions in most part of the world have previously been managed or prevented using phytomedicines (Oniyangi and Cohall, 2018). Medicinal plants have been essential in health management since ancient times (Sofowora et al., 2013). Studies have been carried out globally to evaluate their efficacy and some of the findings have led to the establishment of plant-based medicines (Reiz and Lipp, 1982; Mann et al., 2007; Sofowora et al., 2013; Newman and Cragg, 2020; Adejoh et al., 2021). Availability, affordability, relative safety, and efficacy of natural products have greatly contributed toward their success against some known severe diseases (Oniyangi and Cohall, 2018; Okoh, 2019, Srivastava et al., 2019), for instance, *Camellia sinensis* (L.) Kuntze and *Erigeron breviscapus* (Vaniot) Hand.-Mazz. as neuroprotective agents (López and Calvo, 2011), Ganoderma lucidum and Ganoderma sinense (species of *Ganoderma*) as antitumor agents (Lawal et al., 2019), etc.

The resistance of *Plasmodium falciparum* to chloroquine in the past and to artemisinin and its derivatives currently has attracted worldwide attention. In 2010, the WHO reported a decreased sensitivity of *P. falciparum* to artemisinin and warned of the danger of such resistance (WHO, 2010). This burden of drug resistance on human well-being has drawn the attention of researchers to focus on and devise other therapeutic means using phytomedicine, especially those involving plant bioactive components mediating ligand interactions (Jeong and Ryang, 2019) and gene modification to combat malaria, caused by *Plasmodium* parasite. Several strategies including disruption of feline leukemia virus subgroup C receptor (FLVCR); reduction of FLVCR by gene silencing-techniques; prevention of the interaction between *Plasmodium* thrombospodin related anonymous protein (TRAP) and the Anopheles Saglin protein; prevention of the interaction of surface enolase and plasminogen of mammalian blood meal were suggested to be useful technique for the control of malaria by blocking *Plasmodium* transmission (Adejoh et al., 2018). Recent review also reported some plants belonging to the family of *Violaceae, Rubiaceae,* Cucurbitaceae*,* Poaceae*, Asterids, Rosids*, and *Monocots* with cyclotide antimicrobial peptides, which possess structural similarities to SM1 peptide and were considered as a novel competitive inhibitor of *Plasmodium* TRAP-anopheles saglin binding (Adejoh et al., 2018). Azadirachtin, a bioactive component of *Azadirachta indica* A. Juss. seed extract, was identified to possess structural similarities to artemisinin, a sesquiterpene lactone containing an unusual peroxide-bridge, thought to enhance the anti-plasmodial medicinal characteristic (Brown, 2006; Adejoh et al., 2018). This peroxide bridge is believed to be responsible for the mechanism of action of artemisinin (Adejoh et al., 2018).

Herein, our focus is understanding the complex life cycle of mosquito malaria transmission (both exo- and endo-erythrocytic); their involvement in cerebral malaria via synaptic binding simulation; and relate this with phytochemical properties of the plant (Neem) currently used in sSA to reduce the malaria scourge. Kasaragod et al. (2019) in his studies demonstrated that artemisinin antimalarial drug binds to gephyrin at the same active site where the receptor interaction occurs. Following this indication, we selected Gepherin E as the target towards establishing if any mechanistic similarity exists between these two important natural bioactive molecules.

Bearing in mind the links between medicinal plants and successful anti-malarial drug discovery, we compared the binding affinity of artesunate and azadirachtin to Gephyrin E active site, using molecular dynamic (MD) simulation and the GRIP docking, which enabled more detailed analyses of interaction at the atomic level as compared to the binding free energy estimation from the molecular mechanics/Poisson-Boltzmann surface area (MM/PBSA). Our results lead credence that the bioactive component of the plant Neem can be exploited in pharmaceutical industries for anti-plasmodial drug production.

## Methodology

### Molecular Dynamic Simulation

#### Starting Structures Preparation and MD Simulation

The Gephyrin E domain structures were retrieved from the Protein Data Bank with PDB 1D: 6FGC. The co-crystallized molecules were deleted and any missing residues were added with the aid of modeller (Eswar et al., 2007). B3LYP/6-311++G (d, p) (Jorgensen et al., 1983) level of Gaussian 16 (Weedbrook et al., 2012) were employed to achieve ligand optimization. Following, molecular docking was carried out using the optimised structures with the aid of UCSF Chimera (Yang et al., 2012). FF14SB module (David, 2012; Salomon-ferrer et al., 2012; Soremekun et al., 2019a) of the AMBER forcefield was employed in carrying out MD simulation. The General Amber Force Field (GAFF) and Restrained Electrostatic Potential (RESP) were used in describing the atomic charges of the ligands. Leap variant present in Amber 14 was used for system neutralization and hydrogen atoms addition (Salomon-ferrer et al., 2012; Akinsiku et al., 2020). Following similar protocol earlier reported (Soremekun et al., 2019b; Akinsiku et al., 2020), the system was kept solvated with an orthorhombic box of TIP3P water molecules surrounding all protein atoms at a distance of 9 Å (Jorgensen et al., 1983; Soremekun et al., 2019a). System minimization was carried out first with a 2000 steps minimization using a restraint potential of 500 kcal/mol. Second, we used a 1,000 steps full minimization process without restrain, and afterwards, the system was gradually heated at a temperature of -273.15–26.85°C at 50 ps for simulation time. The system solutes are kept at a potential harmonic restraint of 10 kcal mol 1Å −2 and collision frequency of 1.0 ps-1. Equilibration succeeded heating at an estimate of 500 ps of each system. Temperature at 26.85°C, number of atoms, and pressure at 1bar (isobaric-isothermal ensemble, NPT using Berendsen barostat) were all kept constant. The simulation time was set at 200 ns with each SHAKE algorithm to narrow the hydrogen atom bonds. Each step of the simulation was run for 2fs and an SPFP precision model was adopted. The simulations were kept at constant temperature and pressure (NPT), and Langevin thermostat at collision frequency of 1. ops-2. PTRAJ variant of Amber14 was adopted for further analysis which included root-mean-square deviation (RMSD), root-mean-square fluctuation (RMSF) and Radius of Gyration (Roe and Cheatham, 2013). The data plots were then made with ORIGIN analytical tool and visualization done using UCSF Chimera (Pettersen et al., 2004).

#### Binding Free Energy Estimation

The Molecular Mechanics/Poisson-Boltzmann Surface Area (MM/PBSA) was employed in the estimation of differential binding of Artesunate and Azadirachtin to Gephyrin E (Kollman et al., 2000). MM/PBSA is an end-point energy estimation used in the prediction of binding affinities of ligands and their corresponding protein target. MM/PBSA is mathematically described as:

$$\Delta {\text{G}}_{\text{bind}}{\;=\text{ G}}_{\text{complex}}{(\text{G}}_{\text{receptor}}{\;+\text{ G}}_{\text{inhibitor}})$$ (1)

$$\Delta {\text{G}}_{\text{bind}}\;=\;\Delta {\text{G}}_{\text{gas}}\;+\;\Delta {\text{G}}_{\text{sol }}-\text{ T}\Delta \text{S}$$ (2)

$$\Delta {\text{G}}_{\text{gas}}\;=\;\Delta {\text{E}}_{\text{int}}\;+\;\Delta {\text{E}}_{\text{ele}}\;+\;\Delta {\text{E}}_{\text{vdW}}$$ (3)

$$\Delta {\text{G}}_{\text{sol}}\;=\;\Delta {\text{G}}_{\text{ele,sol}\left(\text{GB}\right)}\;-\;\Delta {\text{G}}_{\text{np,sol}}$$ (4)

$$\Delta {\text{G}}_{\text{np,sol}}\;=\;\gamma \text{SASA }+\;\beta$$ (5)

∆G_gas_ represents the total gas phase energy calculated by intermolecular energy (∆E_int_), electrostatic energy (∆E_elel_), and van der Waals energy (∆E_vdW_). ∆G_sol_ represent the solvation energy, T∆S represent entropy change. ∆G_ele,sol(PB)_ describes polar desolvation energy, while ∆G_np,sol_ describes the non-polar desolvation energy. γ is the surface tension proportionality constant and is set to 0.0072 kcal/(mol-1. Å-2), β is a constant equal to 0, and SASA is the solvent accessible surface area (Å2).

### GRIP Docking Methodology

Vlife^®^ Molecular Design Suite (MDS) 4.6 (Vlife Science Technologies Pvt. Ltd., Pune, India, www.vlifesciences.com) is a robust, modularly multifunctional, and easy to use software suite for Computer-Aided Drug Designing (CADD) (Singla and Bhat, 2010; Igoli J. O. et al., 2014; Pokuri et al., 2014; Singla, 2015; Singla et al., 2016; Sahu et al., 2017; Singla et al., 2017; Singla et al., 2018; Srivastava et al., 2018; Singla and Dubey, 2019; Joon et al., 2021). The structures of the artesunate and azadirachtin were retrieved from PubChem and redrawn using ChemDraw Ultra 8.0 (PerkinElmer LAS [United Kingdom] Ltd., Seer Green, Beaconsfield, Bucks HP9 2FX England) as mol file. After structure preparation, cleaning, and energy optimization, both these ligands were docked in different cavities of the 6FGC. In fact, the X-ray structure of Gephyrin E domain, i.e., 6FGC was cleaned and optimized prior to the docking procedure, and apo_snapshot1 version was used in this study. GRIP docking study was performed on all the eight hydrophobic cavities and tested the affinity of both these ligands for comparison. The parameters used while performing docking simulation were: number of placements: 100; rotation angle: 10°C; exhaustive method; ligand flexible and ligand wise results: 20; scoring function: PLP score. The specific best pose of each ligand respective for each cavity was then processed for the interactive analysis to evaluate van der Waal’s interactions, hydrogen bonding, hydrophobic, pi-staking/aromatic, and charge interactions between ligand and amino acid residues of the hydrophobic cavities (Igoli J. O. et al., 2014; Igoli N. P. et al., 2014; Singla, 2015; Singla et al., 2016; Sahu et al., 2017; Singla et al., 2018; Singla and Dubey, 2019).

Further, to understand the interactions better, especially Van der Waals interactions and hydrophobic interactions, an empirical approach viz. Smart Docking Interaction Calculation Scoring (SDICS) Methodology has been devised, which was basically classifying the interactions into different levels. Methodology was devised on the basis of knowledge and experience gained so far. These are: Weak Van der Waal’s Interaction (V_w_): 1–5 bonding; Moderate Van der Waal’s Interaction (V_m_): 6–10 bonding; Strong Van der Waal’s Interaction (V_s_): 11–20 bonding; Extraordinary Strong Van der Waal’s Interaction (V_x_): ≥21; Weak Hydrophobic Interaction (H_w_): 1–3; Moderate Hydrophobic Interaction (H_m_): 4–7; Strong Hydrophobic Interaction (H_s_): 8–14; and Extraordinary Strong Hydrophobic Interactions (H_x_): ≥15 (Singla et al., 2021).

## Results

### Molecular Dynamics Simulations

**FIGURE 1:**
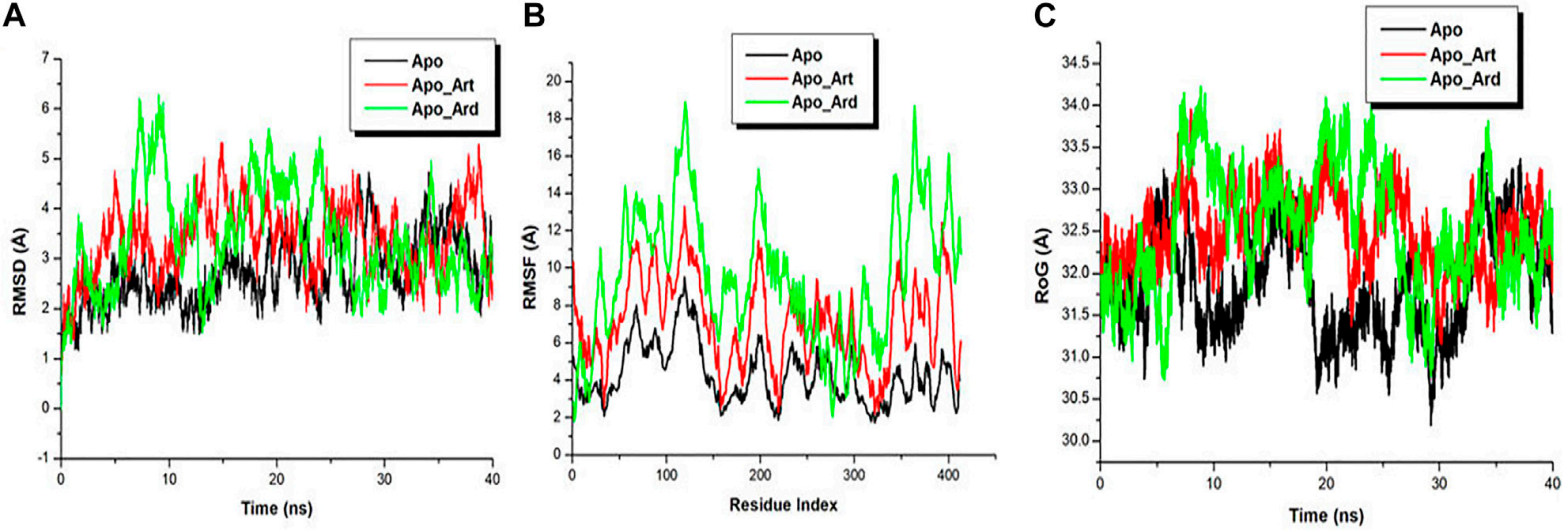
Conformational analysis plot showing stability and atomistic motions among Apo (**black**), Apo_Art (**red**), and Apo_Ard (**green**) systems **(A)**. C-α RMSF plot showing the residual fluctuation of Apo (**black**), Apo_Art (**red**), and Apo_Ard (**green**) systems **(B)**. RoG plot showing the residual compactness of Apo (**black**), Apo_Art (**red**), and Apo_Ard (**green**) systems **(C)**.

MD simulations were conducted to gain insights into the structural changes upon the binding of Artesunate and Azadirachtin in the ligand-binding pocket of Gephyrin E. All produced trajectory during the simulation run were observed for stability and fluctuation. Root mean square deviation (RMSD) and root mean square fluctuation (RMSF) were calculated for the three systems to determine their individual energetic stability and spatial residual fluctuation. The RMSDs of all the backbone atoms of the mutant and wild protein (Figure 1A), as well as the C-α atoms for the residues of the active site, i.e., residues within 5 Å around the ligand were plotted. Figure 1A shows that the three systems reached a convergence as early as 10 ns., indicating the three systems attained stability, hence, a good system for further analysis. Averagely, the RMSD plot revealed that the Apo system exhibited low translational movement and convergence when compared to the Apo-Art and Apo-Ard systems. Furthermore, for a deep insight into the binding of Artesunate and Azadirachtin in the ligand-binding pocket of Gephyrin E, RMSF was used to plot the residual fluctuations during the MD simulation. Figure 1B showed that the Apo-Ard system fluctuates more when compared to the Apo-Art and the Apo system, indicating that Azadirachtin increases the motional movement of the protein when compared to Artesunate. A similar trend was observed in the RoG plot.

**FIGURE 2:**
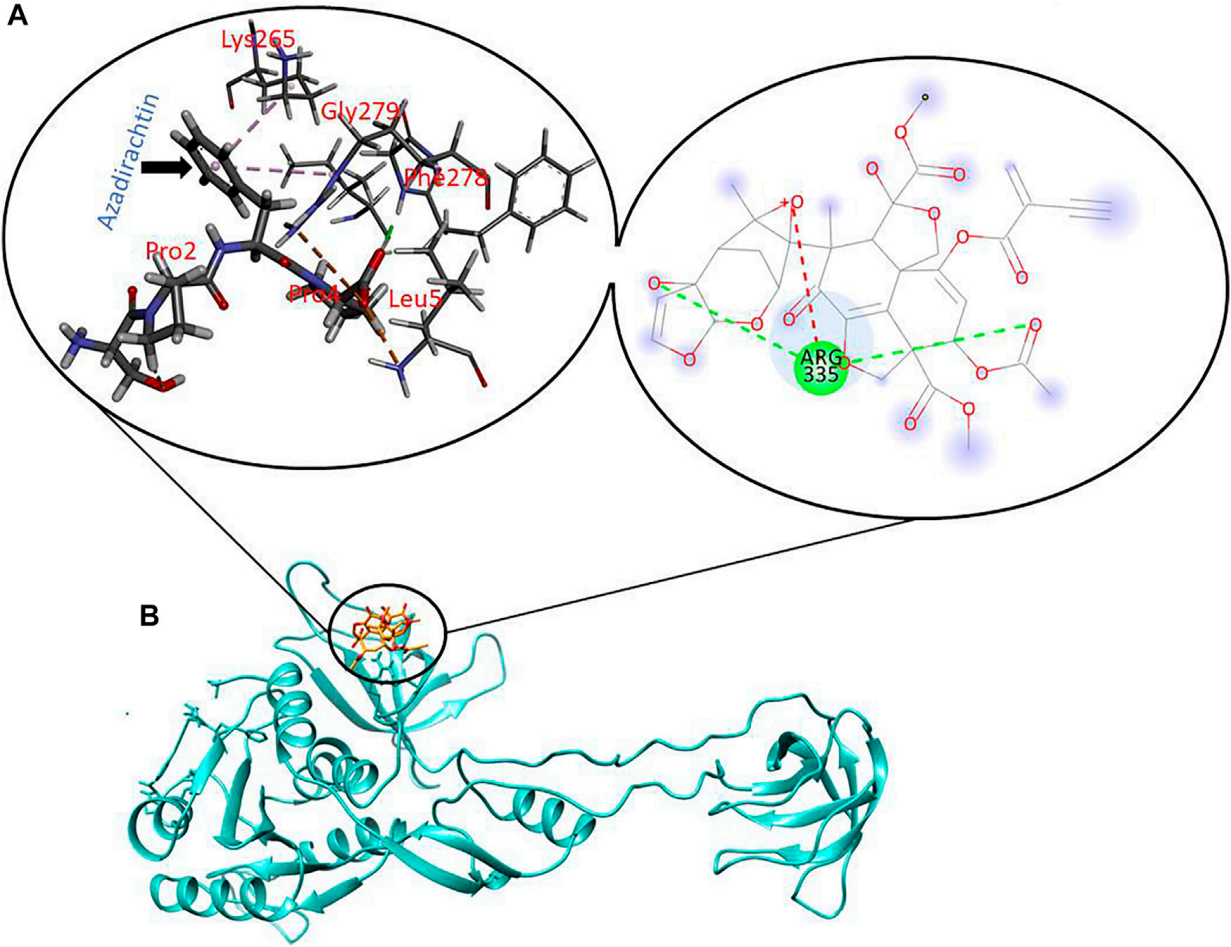
Molecular interactions between key residues and reactive moieties in Gephyrin **(A)**. 3D structure of Azadirachtin in the active site of Gephyrin **(B)**.

**FIGURE 3:**
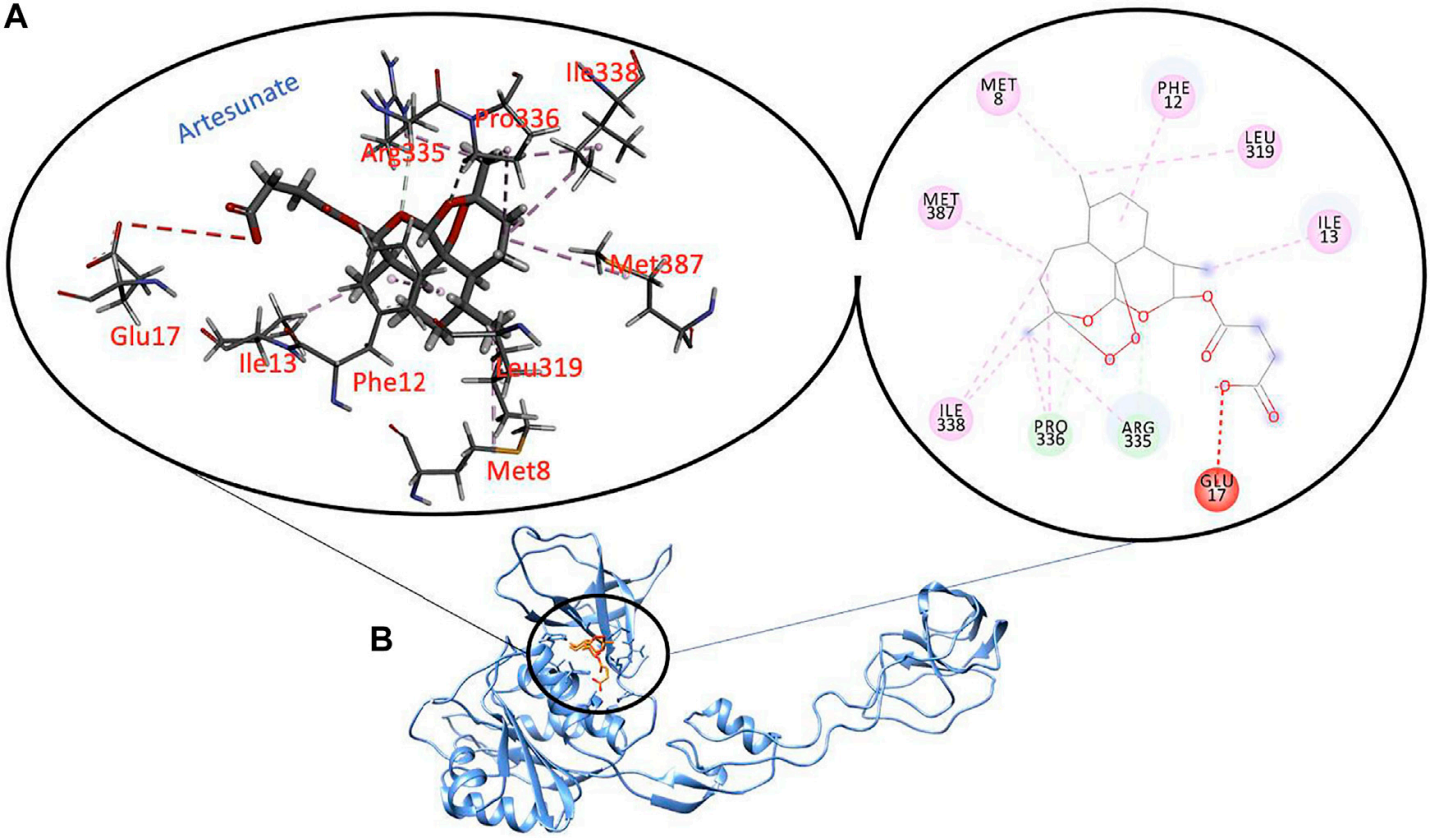
Molecular interactions between key residues and reactive moieties in Gephyrin **(A)**. 3D structure of Artesunate in the active site of Gephyrin **(B)**.

To further explore the binding of Artesunate and Azadirachtin in the ligand-binding pocket of Gephyrin, we used MM/PBSA to explore the binding strength and affinity. The estimation of this binding free energy can help provide insights into the inhibitory mode of Artesunate and Azadirachtin. Our estimations reveal that Azadirachtin had a total binding energy of −36.97 kcal/mol, while Artesunate had a binding energy of -35.73 kcal/mol, suggestive of a better binding affinity of Azadirachtin relative to Artesunate. Comparatively, from Figures 2, 3, the strong binding affinity of Azadirachtin when compared to Artesunate could be corroborated by the strong interactions exhibited between Azadirachtin and the residues present in the active site of Gephyrin E. Most prominent of these interactions are pi-pi alkyl, hydrogen bonds, and covalent interactions. The energy contributions of the active site residues of Gephyrin E, Artesunate, and Azadirachitin are herein presented in Table 1.

**TABLE 1:** Energy contributions between the active site residues of Gephyrin E and Artesunate and Azadirachitin.

Residue	van der Waals (kcal/mol)		Electrostatics (kcal/mol)		Polar Solvation kcal/mol		Non-Polar Solvation kcal/mol	
	Art	Aza	Art	Aza	Art	Aza	Art	Aza
Met8	−0.966	−0.066	−0.179	−2.704	0.529	2.526	−0.089	−0.002
Asp9	−1.180	−0.530	−18.656	187.961	19.203	−184.726	−0.197	−0.142
Phe12	−3.417	−0.294	−0.021	−5.240	0.037	5.349	−0.252	−0.074
Ile13	−1.360	−0.119	0.549	−3.347	−0.475	3.396	−0.254	−0.018
Leu16	−0.624	−0.036	1.124	−4.754	−1.027	4.722	−0.084	−0.000
Arg335	−2.996	−0.056	20.087	−292.239	−19.581	275.218	−0.446	−0.642
Pro336	−1.374	−0.142	−0.733	10.787	1.083	−10.464	−0.107	−0.009
Ile338	−0.694	−0.838	0.209	−1.433	−0.165	1.411	−0.161	−0.203
Tyr355	−0.538	−0.038	0.107	−4.308	0.412	4.342	−0.066	−0.000
MET 387	−0.798	−0.167	−0.201	0.828	0.356	−0.745	−0.152	−0.016

For the validation of the GRIP docking protocol, the co-crystallized ligand artesunate was extracted from 6FGC, and then re-docked as test ligand in the same cavity. Similar dock score obtained when the co-crystallized artesunate was docked as test ligand, which validated the reliability of the docking protocol. For artesunate and azadirachtin’s docking studies, cleaned and optimized apo_snapshot1 version of 6FGC was used. The results from the GRIP docking analysis, tabulated in Table 2, unlike the buck estimate using MM/PBSA, show considerable evidence that artesunate comparatively has more binding affinity to Gephyrin E cavity than azadirachtin, not only for a single cavity, but found to have high multisite potential. Thus, artesunate has more chances to overcome the drug resistance problem, as it is not a highly site-specific drug molecule. The binding affinities of artesunate for different cavities in Gephyrin are in the following order: Cavity 3 > Cavity 2 > Cavity 8 > Cavity 6 > Cavity 4 > Cavity 7 > Cavity 5 > Cavity 1, while in the case of azadirachtin, the binding affinity is in the order: Cavity 6 > Cavity 3 > Cavity 8 > Cavity 2 > Cavity 4. Grip docking results indicated that for cavity 1, cavity 5, and cavity 7, azadirachtin didn’t possess significant binding affinity. One surprising note, for all these 3 cavities, azadirachtin had much stronger interactions with amino acid residues as compared to rest of the cavities (maybe due to the fluid motional movement as indicated Figure 1B), and even surpassed artesunate in some cases. Herein (Table 2), we discuss the interactions in details for those cavities, where the ligands had binding affinity value in the negative.

**TABLE 2:** Grip docking-based interactions study of artesunate and azadirachtin with all the cavities of cleaned and optimized Gephyrin E domain (6FGC), apo_snapshot1. HID: Histidine with hydrogen on the delta nitrogen; HIE: Histidine with hydrogen on the epsilon nitrogen.

S.No	Grip Docking Based Interactions		
	Ligand	Dock Score	Interactions
**Cavity 1**	***Artesunate***	−39.07	**VDW:** Glu218 (**V**_**s**_), HID219 (**V**_**s**_), Arg326 (**V**_**w**_), Thr337 (**V**_**m**_), Val408 (**V**_**s**_), Ile409 (**V**_**w**_), Gly410 (**V**_**s**_), Arg411 (**V**_**x**_), Leu412 (**V**_**w**_)
			**HYI:** Glu218 (**H**_**w**_), HID219 (**H**_**m**_), Val322 (**H**_**w**_), Thr337 (**H**_**m**_), Val408 (**H**_**x**_), Ile409 (**H**_**w**_), Gly410 (**H**_**m**_), Arg411 (**H**_**s**_), Leu412 (**H**_**w**_)
			**CI:** Glu218
			**HB:** Arg411
	***Azadirachtin***	137.22	**VDW:** Glu218 (**V**_**x**_), HID219 (**V**_**x**_), Arg326 (**V**_**s**_), Thr337 (**V**_**s**_), Asn383 (**V**_**w**_), Val408 (**V**_**x**_), Ile409 (**V**_**m**_), Gly410 (**V**_**x**_), Arg411 (**V**_**x**_), Leu412 (**V**_**s**_)
			**HYI:** Glu218 (**H**_**s**_), HID219 (**H**_**x**_), Val322 (**H**_**w**_), Thr337 (**H**_**s**_), Val408 (**H**_**x**_), Ile409 (**H**_**m**_), Gly410 (**H**_**s**_), Arg411 (**H**_**x**_), Leu412 (**H**_**m**_)
			**HB:** Arg326, Gly410, Arg411 (Strong)
**Cavity 2**	***Artesunate***	−65.96	**VDW:** Lys284 (**V**_**s**_), Pro285 (**V**_**s**_), Val311 (**V**_**m**_), Val315 (**V**_**w**_), Glu354 (**V**_**w**_), HID356 (**V**_**x**_), Arg357 (**V**_**s**_), Thr374 (**V**_**w**_)
			**HYI:** Lys284 (**H**_**s**_), Pro285 (**H**_**s**_), Val311 (**H**_**m**_), Val315 (**H**_**w**_), HID356 (**H**_**s**_), Arg357 (**H**_**s**_)
			**HB:** Arg357
	***Azadirachtin***	−24.34	**VDW:** Leu343 (**V**_**w**_), Cys345 (**V**_**w**_), Val347 (**V**_**w**_), HID356 (**V**_**s**_), Ser373 (**V**_**s**_), Thr374 (**V**_**m**_), Gly375 (**V**_**w**_), Leu386 (**V**_**w**_)
			**HYI:** Cys345 (**H**_**w**_), HID356 (**H**_**s**_), Ser373 (**H**_**s**_), Thr374 (**H**_**m**_), Gly375 (**H**_**w**_), Leu386 (**H**_**w**_)
**Cavity 3**	***Artesunate***	−68.27	**VDW:** Met8 (**V**_**m**_), Asp9 (**V**_**m**_), Phe12 (**V**_**s**_), Leu319 (**V**_**w**_), Pro336 (**V**_**s**_), Pro353 (**V**_**m**_), Tyr355 (**V**_**m**_), Met387 (**V**_**s**_), Pro389 (**V**_**w**_), Met407 (**V**_**w**_)
			**HYI:** Met8 (**H**_**m**_), Asp9 (**H**_**m**_), Phe12 (**H**_**w**_), Leu319 (**H**_**w**_), Arg335 (**H**_**w**_), Pro336 (**H**_**s**_), Pro353 (**H**_**m**_), Met387 (**H**_**s**_), Pro389 (**H**_**w**_), Asp405 (**H**_**w**_), Met407 (**H**_**w**_)
			**HB:** Tyr355
	***Azadirachtin***	−53.91	**VDW:** Met8 (**V**_**s**_), Asp9 (**V**_**s**_), Phe12 (**V**_**m**_), Leu319 (**V**_**w**_), Pro336 (**V**_**w**_), Pro353 (**V**_**s**_), Tyr355 (**V**_**m**_), Met387 (**V**_**m**_), Leu388 (**V**_**w**_), Pro389 (**V**_**s**_), Pro390 (**V**_**m**_), Asp405 (**V**_**w**_)
			**HYI:** Met8 (**H**_**s**_), Asp9 (**H**_**s**_), Phe12 (**H**_**w**_), Leu319 (**H**_**w**_), Arg335 (**H**_**w**_), Pro336 (**H**_**m**_), Pro353 (**H**_**s**_), Met387 (**H**_**m**_), Leu388 (**H**_**w**_), Pro389 (**H**_**m**_), Pro390 (**H**_**m**_), Asp405 (**H**_**w**_), Met407 (**H**_**w**_)
**Cavity 4**	***Artesunate***	−57.34	**VDW:** Glu191 (**V**_**s**_), Ile204 (**V**_**m**_), Gly254 (**V**_**w**_), Gly255 (**V**_**w**_), Val256 (**V**_**w**_), Ser257 (**V**_**s**_), Gly259 (**V**_**m**_), Lys261 (**V**_**s**_), Asp262 (**V**_**w**_), Gly308 (**V**_**s**_)
			**HYI:** Glu191 (**H**_**m**_), Leu192 (**H**_**w**_), Ile204 (**H**_**m**_), Gly254 (**H**_**m**_), Gly255 (**H**_**w**_), Val256 (**H**_**w**_), Ser257 (**H**_**w**_), Gly259 (**H**_**w**_), Lys261 (**H**_**s**_), Gly308 (**H**_**m**_)
			**CI:** Glu260, Asp262
			**HB:** Lys261
	***Azadirachtin***	−14.34	**VDW:** Asn190 (**V**_**w**_), Glu191 (**V**_**x**_), Leu192 (**V**_**w**_), Gly202 (**V**_**w**_), Lys203 (**V**_**w**_), Ile204 (**V**_**x**_), Asp231 (**V**_**w**_), Ser257 (**V**_**w**_), Gly259 (**V**_**w**_), Lys261 (**V**_**s**_), Asp262 (**V**_**w**_), Gly308 (**V**_**w**_)
			**HYI:** Asn190 (**H**_**w**_), Glu191 (**H**_**x**_), Leu192 (**H**_**w**_), Lys203 (**H**_**w**_), Ile204 (**H**_**x**_), Ser257 (**H**_**w**_), Gly259 (**H**_**w**_), Lys261 (**H**_**m**_)
			**CI:** Glu191
**Cavity 5**	***Artesunate***	−51.70	**VDW:** Pro4 (**V**_**s**_), Thr6 (**V**_**w**_), Lys10 (**V**_**m**_), Thr14 (**V**_**m**_), Met18 (**V**_**m**_), Gln275 (**V**_**s**_), Ile276 (**V**_**w**_), HIE277 (**V**_**x**_)
			**HYI:** Pro4 (**H**_**s**_), Thr90C (**H**_**w**_), Lys10 (**H**_**s**_), Ile13 (**H**_**w**_), Thr14 (**H**_**m**_), Met18 (**H**_**m**_), Gln275 (**H**_**s**_), HIE277 (**H**_**x**_)
			**HB:** Thr14
	***Azadirachtin***	17.04	**VDW:** Pro4 (**V**_**x**_), Leu5 (**V**_**w**_), Thr6 (**V**_**x**_), Lys10 (**V**_**m**_), Thr14 (**V**_**x**_), Glu17 (**V**_**w**_), Met18 (**V**_**m**_), Ile276 (**V**_**w**_), HIE277 (**V**_**x**_), Phe278 (**V**_**m**_)
			**HYI:** Pro4 (**H**_**s**_), Leu5 (**H**_**w**_), Thr6 (**H**_**x**_), Lys10 (**H**_**m**_), Ala11 (**H**_**w**_), Thr14 (**H**_**s**_), Met18 (**H**_**w**_), HIE277 (**H**_**s**_), Phe278 (**H**_**m**_)
			**CI:** HIE277
			**HB:** Thr6
**Cavity 6**	***Artesunate***	−60.65	**VDW:** Phe3 (**V**_**w**_), Val256 (**V**_**w**_), Ser257 (**V**_**s**_), Met258 (**V**_**x**_), Gly259 (**V**_**w**_), Glu260 (**V**_**s**_), Asp262 (**V**_**w**_), Lys265 (**V**_**s**_), Gln266 (**V**_**w**_), Arg280 (**V**_**s**_)
			**HYI:** Phe3 (**H**_**w**_), Ser257 (**H**_**w**_), Met258 (**H**_**s**_), Glu260 (**H**_**m**_), Asp262 (**H**_**w**_), Lys265 (**H**_**m**_), Arg280 (**H**_**s**_), Pro288 (**H**_**w**_)
			**HB:** Lys265
	***Azadirachtin***	−59.87	**VDW:** Phe3 (**V**_**m**_), Ser257 (**V**_**w**_), Met258 (**V**_**s**_), Gly259 (**V**_**w**_), Glu260 (**V**_**w**_), Asp262 (**V**_**w**_), Lys265 (**V**_**s**_), Gln266 (**V**_**m**_), Arg280 (**V**_**x**_), Leu287 (**V**_**w**_)
			**HYI:** Pro2 (**H**_**w**_), Phe3 (**H**_**w**_), Met258 (**H**_**s**_), Asp262 (**H**_**w**_), Lys265 (**H**_**m**_), Arg280 (**H**_**m**_), Leu287 (**H**_**w**_)
			**HB:** Gly259, Lys265, Gln266
**Cavity 7**	***Artesunate***	−53.94	**VDW:** Val21 (**V**_**s**_), Thr24 (**V**_**w**_), Ala38 (**V**_**s**_), Lys155 (**V**_**w**_), Asn178 (**V**_**m**_), Lys327 (**V**_**m**_), Gly330 (**V**_**m**_), Ile331 (**V**_**w**_), Leu332 (**V**_**m**_)
			**HYI:** Val21 (**H**_**s**_), Thr24 (**H**_**m**_), Ala38 (**H**_**s**_), Lys155 (**H**_**w**_), Gly156 (**H**_**w**_), Lys327 (**H**_**m**_), Gly330 (**H**_**m**_), Leu332 (**H**_**s**_)
			**HB:** Asn178, Lys327
	***Azadirachtin***	285.81	**VDW:** Val21 (**V**_**m**_), Leu22 (**V**_**m**_), Gly23 (**V**_**w**_), Thr24 (**V**_**s**_), Arg35 (**V**_**w**_), Val36 (**V**_**x**_), Leu37 (**V**_**s**_), Ala38 (**V**_**x**_), Gln39 (**V**_**w**_), Lys155 (**V**_**x**_), Gly156 (**V**_**x**_), Thr157 (**V**_**w**_), HIE158 (**V**_**m**_), Glu176 (**V**_**s**_), Val177 (**V**_**w**_), Asn178 (**V**_**x**_), Gln329 (**V**_**w**_), Gly330 (**V**_**s**_), Ile331 (**V**_**w**_), Leu332 (**V**_**s**_)
			**HYI:** Val21 (**H**_**s**_), Leu22 (**H**_**w**_), Gly23 (**H**_**w**_), Thr24 (**H**_**s**_), Val36 (**H**_**x**_), Leu37 (**H**_**m**_), Ala38 (**H**_**x**_), Gln39 (**H**_**w**_), Lys155 (**H**_**x**_), Gly156 (**H**_**s**_), Glu176 (**H**_**w**_), Val177 (**H**_**w**_), Asn178 (**H**_**s**_), Gly330 (**H**_**m**_), Ile331 (**H**_**w**_), Leu332 (**H**_**s**_)
			**HB:** Val36, Gln39, Gly156
**Cavity 8**	***Artesunate***	−64.49	**VDW:** Pro48 (**V**_**w**_), Pro49 (**V**_**s**_), Phe50 (**V**_**s**_), Ala52 (**V**_**w**_), Ala77 (**V**_**w**_), Gly78 (**V**_**s**_), Glu79 (**V**_**w**_), Gly96 (**V**_**m**_), Ala97 (**V**_**w**_), Pro98 (**V**_**s**_)
			**HYI:** Pro48 (**H**_**m**_), Pro49 (**H**_**x**_), Phe50 (**H**_**w**_), Ala52 (**H**_**m**_), Ala77 (**H**_**s**_), Gly78 (**H**_**s**_), Glu79 (**H**_**w**_), Gly96 (**H**_**w**_), Ala97 (**H**_**w**_), Pro98 (**H**_**x**_)
			**HB:** Gly78
	***Azadirachtin***	−40.42	**VDW:** Pro48 (**V**_**w**_), Pro49 (**V**_**s**_), Ala77 (**V**_**s**_), Gly78 (**V**_**x**_), Gly96 (**V**_**w**_), Pro98 (**V**_**w**_)
			**HYI:** Leu47 (**H**_**w**_), Pro49 (**H**_**m**_), Ala77 (**H**_**s**_), Gly78 (**H**_**s**_)
			**HB:** Gly96

***Keys***: VDW: Van der Waal`s interactions; HYI: Hydrophobic interactions; CI: Charge interactions; HB: Hydrogen bonding. V_w_: Weak Van der Waal’s Interaction; V_m_: Moderate Van der Waal’s Interaction; V_s_: Strong Van der Waal’s Interaction; V_x_: Extraordinary Strong Van der Waal’s Interaction; H_w_: Weak Hydrophobic Interaction; H_m_: Moderate Hydrophobic Interaction; H_s_: Strong Hydrophobic Interaction; H_x_: Extraordinary Strong Hydrophobic Interactions.

Succinctly, for cavity 1, artesunate had significant Van der Waal’s interactions with Glu218, HID219, Val408, Gly410, and Arg411 while having significant hydrophobic interactions with Val408 and Arg411 only. Apart from this, 18C of artesunate was having charge interaction with Glu218 at a bond distance of 3.740 Å while the 27O of artesunate exhibited hydrogen bonding with Arg411 at a bond distance of 2.190 Å. Azadirachtin interactions with amino acid residues of Gephyrin E’s cavity 1 were better than artesunate, but the binding affinity was in positive range, hence, not discussed here.

For the cavity 2, artesunate was having significant Van der Waal’s and hydrophobic interactions with Lys284, Pro285, HID356, and Arg357 amino acid residues of 6FGC. Apart from these, 26O of artesunate was having hydrogen bonding with Arg357. On the other hand, azadirachtin had some significant Van der Waal’s and hydrophobic interactions with HID356 and Ser373. No other interactions apart from Van der Waal’s and hydrophobic interactions were found for azadirachtin.

Similarly, for cavity 3, artesunate had some significant Van der Waal’s interactions with Phe12, Pro336, and Met387 while having significant hydrophobic interactions with Pro336 and Met387 only. Apart from these, 24O of artesunate had hydrogen-bonding interaction with Tyr355 of 6FGC. Azadirachtin, on the other hand, had some significant Van der Waal’s interactions with Met8, Asp9, Pro353, and Pro389 and significant hydrophobic interactions with Met8, Asp9, and Pro353 amino acid residues in cavity 3 of 6FGC. No other interactions apart from Van der Waal’s and hydrophobic interactions were found in case of azadirachtin.

In case of the cavity 4, artesunate was having significant Van der Waal’s interactions with Glu191, Ser257, Lys261, and Gly308 while having significant hydrophobic interactions with Lys261 only. Moreover, 18C of artesunate was having charge interactions with Glu260 and Asp262 at a bond distance of 4.825 and 4.142 Å, respectively. Further, 26O of artesunate was exhibiting hydrogen bonding with Lys261 at a bond distance of 2.087 Å. On the other hand, azadirachtin was having significant Van der Waal’s interactions with Glu191, Ile204, and Lys261 while having significant hydrophobic interactions with Glu191 and Ile204 only. Apart from these, 21O of azadirachtin was having charge interaction with Glu191 at a bond distance of 4.506 Å.

Moreover, in the case of cavity 5, artesunate had some significant Van der Waal’s interactions with Pro4, Gln275, and HIE277 while having strong hydrophobic interactions with Pro4, Lys10, Gln275, and HIE277 amino acid residues of 6FGC. Apart from these, 27O of artesunate was having hydrogen bonding with Thr14 at a bond distance of 2.153 Å. Azadirachtin interactions with amino acid residues were found to be significant, but since the binding affinity was in positive range, it is not discussed here.

In case of the cavity 6, artesunate was having significant Van der Waal’s interactions with Ser257, Met258, Glu260, Lys265, and Arg280 while having significant hydrophobic interactions with Met258 and Arg280 only. Apart from these, 23O of artesunate was having hydrogen bonding with Lys265 at a bond distance of 2.371 Å. On the other hand, azadirachtin was having significant Van der Waal’s interactions with Met258, Lys265, and Arg280 while having significant hydrophobic interactions with Met258 only. Moreover, 31H of azadirachtin was having hydrogen bonding with Gly259 at a bond distance of 2.001 Å, 25O of this ligand was having hydrogen bonding with Lys265 at a bond distance of 2.564 Å, while 27O of azadirachtin was exhibiting hydrogen bonding with Gln266 at a bond distance of 1.697 Å.

For the cavity 7, artesunate was having significant Van der Waal’s interactions with Val21 and Ala38 only while having significant hydrophobic interactions with Val21, Ala38, and Leu332 amino acid residues of 6FGC. Apart from that, 20O and 26O of artesunate were having hydrogen bonding with Asn178 and Lys327 at a bond distance of 2.251 and 2.111 Å, respectively. Azadirachtin interactions with amino acid residues were better, but since the binding affinity was in positive range, we will not discuss it here.

In the case of cavity 8, artesunate was having significant Van der Waal’s interactions with Pro49, Phe50, Gly78, and Pro98 while having significant hydrophobic interactions with Pro49, Ala77, Gly78, and Pro98 amino acid residues of 6FGC. Apart from that, 22O of artesunate was having hydrogen bonding with Gly78 at a bond distance of 2.238 Å. On the other hand, azadirachtin was having significant Van der Waal’s interactions with Pro49, Ala77, and Gly78 while having significant hydrophobic interactions with Ala77 and Gly78 only. Moreover, 31H of azadirachtin was having hydrogen bonding with Gly96 at a bond distance of 2.297 Å.

Though there is marginal difference in the binding affinity for both the ligands in case of cavity 3, cavity 4, cavity 6, and cavity 8, the interactions revealed that azadirachtin was also having a strong potential to act on the residues of 6FGC.

## Discussion and Future Perspectives

Molecular dynamics is a crucial tool in structural molecular biology and computer-aided drug design. In attempts to understand biochemical processes, the combination of both ligand and structure-function-based analysis for drug design approaches remains a promising tool for the discovery and development of new molecules with potential anti-malaria activities (Ojha and Ray, 2015). During malaria parasite invasion of the brain (cerebral malaria), metabolite such as gamma amino butyric acid (GABA) and pipecolate are elevated at the trophozoites and schizont stage (post invasion). *Plasmodium falciparum* invasion of the red blood cells lead to break down of haemoglobin whose globin component is utilized for the synthesis of various *plasmodium* proteins (Beri et al., 2019). *Plasmodium falciparum* can convert alpha ketoglutarate to glutamate, which in turn converted to GABA. In addition, other inflammatory metabolites such as those found in the kynurenine pathway (quinolinic and kynurenic acid) are thought to be important in cerebral malaria pathogenesis. Quinolinic acid has been shown to cause seizures in animal models of brain disease, while kynurenic acid is an antagonist and is generally thought of as neuro-protective (John et al., 2006).

Increase in the secretion of GABA mediated by *P. falciparum* schizont infected erythrocyte is suggestive for the clinical manifestation of a COMA associated with cerebral malaria (Beri et al., 2019) Gephyrin-mediated clustering of GABA_A_ and glycine receptors underlies fast inhibitory signalling at central synapses (Jeong and Ryan, 2019). Kasaragod et al. (2019) in his studies demonstrated that artemisinin antimalarial drug binds to gephyrin at the same active site where the receptor interaction occurs. Neurotransmission inhibition is mediated by synaptic GABA_A_ and glycine receptors in the central nervous system (CNS). Gephyrin is a key protein that reinforces synaptic recruitment of both receptors (Jeong and Ryan, 2019).

Gephyrin is a Greek word which means “bridge” and represents the functional significance of bridging between glycine receptors and the cytoskeleton (Tyagarajan and Fritschy, 2014; Jeong and Ryan, 2019). It is a 93 kDa protein with N-terminal geph G and C-terminal geph E domains, connected through a long unstructured linker often called the geph C domain (Tyagarajan and Fritschy, 2014). These domains play critical roles in complex formation and a not well-understood role in oligomerization to zero in on receptors at synapses.

Previously, *in vitro* analysis had revealed geph G assembles as a trimer and geph E assembles as a dimer, which resembles the unusual disulphide bridge of SM1 peptide (Ghosh et al., 2009; Jeong and Ryan, 2019)). The geph C linker contains post-translational modification sites thought to regulate the formation of gephyrin clusters (Jeong and Ryan, 2019). Among the three domains, the geph E domain is the one that directly interacts with the inhibitory receptors (Jeong and Ryan, 2019). The GABA_A_ and glycine receptors are part of the larger Cys-loop family of pentameric ligand-gated ion channels (Jeong and Ryan, 2019). The Cys-loop family was suggested in recent review (Adejoh et al., 2018) to be the major molecular component responsible for the anti-plasmodial characteristic of phytomedicine, which possess cyclotide antimicrobial peptides. It is thought that all the subunit in the pentamer shares a conserved architecture, including four transmembrane α helices (M1–M4) with a poorly conserved and often large and disordered intracellular loop between M3 and M4 (Jeong and Ryan, 2019). It is this flexible loop that can bind to a groove in the geph E domain of gephyrin (Kim et al., 2006; Maric et al., 2011; Maric et al., 2014; Jeong and Ryan, 2019).

Kasaragod et al. (2019), using the concept of neuro-interaction, identified the artemisinin binding site on gephyrin and provides structural and biochemical insights into the mechanism of artemisinin in gephyrin-mediated inhibitory receptor clustering. Geph E domain as discussed earlier is the target for artemisinin, as reported using a crystallographic approach to define atomic-scale mechanisms of the small molecules (Jeong and Ryan, 2019; Kasaragod et al., 2019). The experimental approach revealed four structures of the geph E domain; two of which were shown to be bounded by the artemisinin, artemether, and artesunate; the other two were bounded by peptides from the intracellular loops of the GABA_A_ α3 and glycine β receptor subunits. Interestingly, the artemisinin-binding pocket overlaps with the receptor binding pocket and shares key points of interaction, implying that these drugs may directly compete with receptor binding (Jeong and Ryan, 2019). The receptor-gephyrin interaction occurs in a large groove formed by geph E subdomains III and IV (Jeong and Ryan, 2019). Both receptor-derived peptides nestle within this hydrophobic groove. The peptides from the GABA_A_ R α3 subunit and GlyR β subunit form key interactions with F330, I331, and R635 in gephyrin. Intriguingly, the two artemisinins are positioned to form interactions with these same residues (Jeong and Ryan 2019). Hypothetically, it is most probable that the process and reports of coma associated with cerebral malaria may be due to the extrusion of GABA and homocysteine by *P. falciparum* schizont-infected erythrocytes. This provides important clinical implications enabling further investigation into bioactive compounds of plants origin with a view to mitigate pathogenesis of malaria in all its forms.

Similarly, Figures 2, 3 present the binding interaction (affinity binding) between artesunate (an Artemisinin derivatives) and azadirachtin to the active site of gephyrin E. The result of computational simulation study shows that azadirachtin has a high binding affinity to the active site of gephyrin when compared to artesunate binding. However, the GRIP docking shows otherwise bearing, artesunate has comparatively more binding affinity to azadirachtin, albeit marginal difference was found in the binding affinity for both the ligands for cavity 3, cavity 4, cavity 6, and cavity 8, with the interactions revealing that azadirachtin has a strong potential to act on the residues of 6FGC.

Results from these disparate methods suggest that azadirachtin properly developed may be as effective an anti-malarial agent as artesunate. Artesunate and azadirachtin binds to the same active site of gephyrin suggesting that both compounds may possess similar structure, side chains, and functionality. The binding of artesunate to gephyrin E reported earlier to stabilize the interaction between GABA_A_ receptors and gephyrin leading to *trans*-differentiation of the α-cells into the β-cells enabling artesunate exhibit its antimalarial activity (Kasaragod et al. 2019). With the similarities between artesunate/azadirachtin as reported in this study, it is most probable also that both metabolites may share same pattern of molecular activities against malaria parasite invasion.

The paucity of literature on risk factors for cognitive impairment as a result of malaria/cerebral malaria highlights the need for additional studies in this area, and also it brings to the fore the need for further studies on phyto-compounds used in combating the scourge of malaria across sSA.

Conclusively, the present study compares the binding affinity of artesunate and azadirachtin a metabolite present in neem plant to the active site of gephyrin, thought to underlie their roles in clustering inhibitory ligand-gated ion channels at synapses (Jeong and Ryan, 2019). The formation of clustering inhibitory ligand-gated ion channels at synapses may be due to the hydrophobic nature of the side chains of glycine and GABA, and this could prevent the transmission of *Plasmodium* parasite across synaptic membrane. As a result of this, parasite anchoring leading to transmembrane differentiation would be truncated, hence, the control of malaria disease. The molecular details provide foundational insights for this study probing mechanisms of receptor clustering which earlier suggests the anti-malaria potential of artemisinin (Maric et al., 2014). The actual roles of artesunate in destabilizing synaptic signalling complexes at concentrations used to treat malaria are less clear (Jeong and Ryan, 2019). Some of the challenges studying the effects of this drug class on neuronal signalling is the documented cytotoxicity in cell culture and animal studies, as well as neurotoxicity in human clinical studies (Efferth and Kaina, 2010; Jeong and Ryan, 2019), and this may probably be due to high levels of extracellular homocysteine, which have been implicated in neurological damage and disrupting the blood brain barrier (Hunt and Grau, 2003; Srivastava et al., 2019). Going forward, it will be exciting to visualize complexes of full receptors with gephyrin to better understand how synaptic anchoring is achieved and how small molecules may destabilize it, leading to the effective control of malaria disease using plant-based drugs/components. It is of note also, in this study, that it was surprising that, for all the 3 (1.5 and 7) cavities, azadirachtin had less binding but much stronger interactions with amino acid residues as compared to the rest of the cavities, and even surpassed artesunate in some cases; this may explain the higher total binding energy from the MD simulation. The GRIP docking enabled a more detailed interaction at the atomic resolution level as compared to the binding free energy estimation from the Molecular Mechanics/Poisson-Boltzmann Surface Area (MM/PBSA). Further, from the GRIP docking result, it is evident that both compounds have more chances to overcome the drug resistance problem, as both are not highly site-specific drug molecules. Moving forward, it is highly essential for the combination of disparate molecular/biophysical tools for attempting rational drug design from natural bioactive compounds.

## Data Availability

The original contributions presented in the study are included in the article/Supplementary Material, and further inquiries can be directed to the corresponding authors.
